# Detection of Medical Misinformation in Hemangioma Patient Education: Comparative Study of ChatGPT-4o and DeepSeek-R1 Large Language Models

**DOI:** 10.2196/76372

**Published:** 2025-11-18

**Authors:** Guoyong Wang, Ye Zhang, Weixin Wang, Yingjie Zhu, Wei Lu, Chaonan Wang, Hui Bi, Xiaonan Yang

**Affiliations:** 1 Department of Hemangioma and Vascular Malformation, Plastic Surgery Hospital, Chinese Academy of Medical Sciences and Peking Union Medical College, 33 Badachu Road, Shijingshan District, Beijing, 100144, China, 86 18810601889, 86 01053968149; 2 Department of Internal Medicine, Plastic Surgery Hospital, Chinese Academy of Medical Sciences and Peking Union Medical College, Beijing, 100144, China

**Keywords:** medical rumors, large language models, hemangioma, semantic similarity, classification performance, artificial intelligence, AI

## Abstract

**Background:**

This study examines the capability of large language models (LLMs) in detecting medical rumors, using hemangioma-related information as an example. It compares the performances of ChatGPT-4o and DeepSeek-R1.

**Objective:**

This study aimed to evaluate and compare the accuracy, stability, and expert-rated reliability of 2 LLMs, ChatGPT-4o and DeepSeek-R1, in classifying medical information related to hemangiomas as either “rumors” or “accurate information.”

**Methods:**

We collected 82 publicly available texts from social media platforms, medical education websites, international guidelines, and journals. Of the 82 items, 47/82 (57%) were labeled as “rumors,” and 35/82 (43%) were labeled as “accurate information.” Three vascular anomaly specialists with extensive clinical experience independently annotated the texts in a double-blinded manner, and disagreements were resolved by arbitration to ensure labeling reliability. Subsequently, these texts were input into ChatGPT-4o and DeepSeek-R1, with each model generating 2 rounds of results under identical instructions. Output stability was assessed using bidirectional encoder representations from transformers–based semantic similarity scores. Classification accuracy, precision, recall, and *F*_1_-score were calculated to evaluate the performance. Additionally, 2 medical experts independently rated the model outputs using a 5-point scale based on clinical guidelines. Statistical analyses included paired *t* tests, Wilcoxon signed-rank tests, and bootstrap resampling to compute confidence intervals.

**Results:**

In terms of semantic stability, the similarity distributions for the 2 models largely overlapped, with no statistically significant difference observed (mean difference=−0.003, 95% CI −0.011 to 0.005; *P*=.30). Regarding classification performance, DeepSeek-R1 achieved higher accuracy (0.963) compared to ChatGPT-4o (0.910), and also performed better in terms of precision (0.978 vs 0.940), recall (0.957 vs 0.894), and *F*_1_-score (0.967 vs 0.916). Expert evaluations revealed that DeepSeek-R1 significantly outperformed ChatGPT-4o on both “rumor” items (mean difference=0.431; *P*<.001; Cohen *d_z_*=0.594) and “accurate information” items (mean difference=0.264; *P*=.045; Cohen *d_z_*=0.352), with a particularly pronounced advantage in rumor detection.

**Conclusions:**

DeepSeek-R1 demonstrated greater accuracy and rationale in detecting medical rumors compared with ChatGPT-4o. This study provides empirical support for the application of LLMs and recommends optimizing accuracy and incorporating real-time verification mechanisms to mitigate the harmful impact of misleading information on patient health.

## Introduction

In recent years, artificial intelligence (AI) has drawn considerable attention in detecting medical and health-related rumors [1,2]. Some studies have conducted systematic reviews on the application of AI technologies, such as text mining and machine learning, for the automatic identification of health misinformation [3]. Nonetheless, recognizing medical rumors remains a challenge due to the scarcity of high-quality specialized datasets and the extensive effort required by medical experts for annotation [4,5], making it difficult to train highly accurate rumor detection models. Moreover, as conversational AI assistants become increasingly integrated with and partially replace traditional search engine functionalities, more individuals are turning to chatbots for medical information [6,7]. However, current large language models (LLMs) lack robust verification mechanisms and often struggle to differentiate genuine from false medical information, frequently producing factually incorrect or imprecise answers—commonly known as “hallucinations” [6,8,9]. In the medical field, the risks posed by misinformation are particularly severe, as misleading content can undermine trust in health care systems, alter treatment decisions, and even lead patients to delay or reject scientifically validated therapies, opting instead for unsupported and potentially harmful treatments [10].

To ground our investigation concretely, we focused on vascular tumors and malformations—a field where rapidly evolving medical classifications often cause significant public confusion and misinformation [11]. The International Society for the Study of Vascular Anomalies classification is continuously updated, with the 2025 edition significantly revising its 2018 predecessor by introducing a new category, potentially unique vascular anomaly, incorporating multiple genetic syndromes into the classification framework, and implementing extensive terminology revisions. Such frequent updates complicate both clinical diagnosis and public comprehension [11,12]. A prominent example is the lesion previously termed “cavernous hemangioma,” which has now been redefined as a subtype of “venous malformation.” However, outdated terminology persists widely in patient forums and online sources, creating a gap between current medical standards and lay perceptions. This misinformation can lead directly to clinical risks, such as misdiagnosis, delayed treatments, or unnecessary interventions, highlighting the critical need to address inaccuracies and outdated information [13].

In this context, our study selected 2 widely adopted conversational AI models—OpenAI’s ChatGPT-4o and the open-source DeepSeek-R1—as research subjects [14,15]. This combination not only represents the 2 primary development trajectories (closed-source versus open-source) of contemporary LLMs but also establishes a baseline task for subsequent benchmarking, allowing future studies to incorporate additional LLMs and facilitate longitudinal comparability. We conducted a classification evaluation of medical statements concerning hemangiomas and vascular malformations, focusing particularly on the models’ ability to identify incorrect medical claims (rumors). By comparing the performance of these 2 models on relevant statements, our research aims to evaluate the current capabilities and limitations of AI models in verifying medical information and to provide insights for enhancing rumor-detection capabilities in medical AI systems in future work.

## Methods

### Study Design and Overview

**Figure 1.**
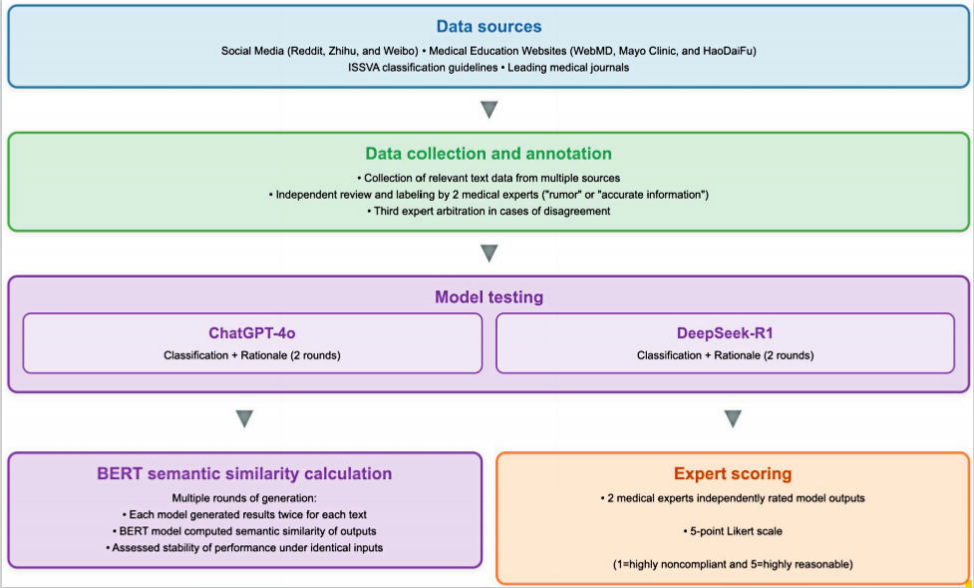
Research methodology framework. BERT: bidirectional encoder representations from transformers; ISSVA: International Society for the Study of Vascular Anomalies.

Our study used publicly available texts from global social media platforms (eg, Reddit, Zhihu, and Weibo); medical education websites (eg, WebMD, Mayo Clinic, and HaoDF or HaoDaifu Online), the International Society for the Study of Vascular Anomalies classification resources, relevant guidelines, and medical journals (Multimedia Appendix 1). In total, 82 statements were collected, with 47 (57%) classified as “rumors” and 35 (43%) as “accurate information.” These statements covered key educational aspects of patients with hemangiomas and vascular malformations, including (1) nomenclature and classification, (2) pathogenesis and natural history, (3) risk stratification and complications, (4) assessment and referral, (5) treatment and peritreatment issues, and (6) prognosis and follow-up. All texts collected were independently reviewed by medical experts and labeled as either “rumors” or “accurate information,” based on guideline-supported factual accuracy. Figure 1 provides an overview of the study workflow.

### Ethical Considerations

This study used only publicly available, nonidentifiable text data and did not involve clinical interventions, access to medical records, or collection of personal identifiers. In accordance with the Measures for the Ethical Review of Life Science and Medical Research Involving Humans, research using lawfully obtained public data or anonymized information may be exempt from ethics review (Article 32). Therefore, an ethics application was not required for this study [16]. Since the data were public and nonidentifiable, informed consent was not required. No compensation was provided to any individuals in relation to this study.

### Data Collection and Annotation

Two medical experts specializing in vascular anomalies (with 5 and 10 y of clinical experience, respectively) independently reviewed and labeled each statement as either “rumor” or “accurate information.” To minimize bias, all items were anonymized by removing source identifiers and engagement metrics prior to labeling, and annotators remained double-blinded to each other’s decisions. In cases of disagreement, arbitration was conducted by a third medical expert with 15 years of clinical experience, resulting in a unified set of labels and ensuring labeling reliability. Potential biases were mitigated through independent dual review, third-party arbitration, and prespecified labeling guidelines.

### Model Testing

After labeling, the texts were input into 2 LLMs—ChatGPT-4o and DeepSeek-R1—for testing. The process is presented in Textbox 1.

Textbox 1. **Model testing process.**

- Prompts and outputs: to minimize bias introduced by variations in prompting and to highlight baseline comparability, both models received the identical concise instruction: “evaluate the following statement for accuracy and reliability in the context of hemangioma and vascular malformation treatment.” Each model classified the texts as either “rumor” or “accurate information,” accompanied by a brief rationale (Multimedia Appendix 2).
- Multiple rounds of generation: to reduce the effects of random output, each model generated results twice for each text. A bidirectional encoder representations from transformers model was then used to compute the semantic similarity of these 2 outputs to assess the stability of the model’s performance under identical inputs.

### Expert Scoring

In addition to classification results, 2 medical experts independently assessed the compliance of each model’s output with clinical guidelines. Evaluations were performed using a 5-point Likert scale (1= highly noncompliant, 5=highly reasonable). The medical experts remained blinded to both the model identities (ChatGPT-4o vs DeepSeek-R1) and each other’s scores. Detailed scoring criteria are provided in Multimedia Appendix 3.

### Statistical Analysis

#### Semantic Similarity and Stability

Semantic stability was assessed by calculating bidirectional encoder representations from transformers (BERT)–based similarity scores between 2 independently generated outputs for each statement (see Multimedia Appendix 4 for detailed code). Descriptive statistics, including means, SDs, medians, and IQRs, were reported. Differences between models were compared using paired Wilcoxon signed-rank tests (due to partially nonnormal distributions). Additionally, 95% bias-corrected and accelerated CIs for mean differences were computed via 10,000 bootstrap resamples to ensure robust interval estimation.

#### Classification Performance

Classification accuracy, precision, recall, and *F*_1_-scores were calculated based on standard definitions, with error distributions visualized using confusion matrices. This approach allows comprehensive evaluation of global and class-specific performance and is particularly suitable for scenarios involving class imbalance.

#### Expert Ratings

Two clinical experts independently provided ratings on a 5-point Likert scale for each of the 82 statements (47 rumors and 35 accurate statements) in 2 separate rounds. The mean rating for each item was computed as the final score. For each model, descriptive statistics such as mean (SD) and 95% CIs were calculated, treating each statement as an independent unit. Between-model comparisons were performed using paired 2-tailed *t* tests (assuming normality of differences) supplemented by Wilcoxon signed-rank tests as a robust alternative, with Cohen *d_z_* effect sizes reported. Within-model comparisons between “rumors” and “accurate information” were conducted using Welch *t* test to account for unequal sample sizes and potential variance heterogeneity. Reviewer agreement and reliability were assessed using Cronbach α and interclass correlation coefficients (ICCs), ICC(2,1)/ICC(2,k). All tests were 2-tailed, with statistical significance defined as *P*<.05.

## Results

### Overview

This study systematically compared the performance of ChatGPT-4o and DeepSeek-R1 in classifying statements related to hemangiomas and vascular malformations across three dimensions: (1) the stability of 2 independent outputs, assessed using BERT-based semantic similarity metrics; (2) classification performance, evaluated by accuracy, precision, recall, and *F*_1_-score; and (3) clinical appropriateness of model outputs as rated by experts on a 5-point scale. For expert ratings, statistical inference was conducted using a paired design with Wilcoxon signed-rank tests, effect sizes (r), and 95% bias-corrected and accelerated CIs.

### Semantic Similarity Analysis

To evaluate the semantic similarity between the model-generated responses, we used a BERT-based scoring approach (detailed in Multimedia Appendix 5). Multimedia Appendix 6 shows the distribution of the scores for ChatGPT-4o and DeepSeek-R1. Overall, the distributions for both models exhibited substantial overlap, with ChatGPT-4o displaying a slightly narrower distribution, while DeepSeek-R1 showed a marginally wider range. During paired comparisons, 1 pair with identical observations was excluded, resulting in 81 (99%) paired samples for analysis. The Wilcoxon signed-rank test indicated no significant difference in stability between the 2 models (W=1440.5; *z*=−1.036; *P*=.30), with a mean difference of only −0.003 (95% bootstrap CI −0.011 to 0.005, r=−0.115) as shown in Table 1. These findings suggest comparable semantic similarity and stability performance between the 2 models.

**Table 1.** Stability comparison between ChatGPT-4o and DeepSeek-R1 based on bidirectional encoder representations from transformers semantic similarity scores.^a^

Model and comparison	Mean (SD)	Median (IQR)	Range
ChatGPT-4o (N=82)	0.9000 (0.0250)	0.9060 (0.8870‐0.9180)	0.8250‐0.9400
DeepSeek-R1 (N=82)	0.8970 (0.0320)	0.9010 (0.8850‐0.9140)	0.7800‐1.0000

a Paired difference (DeepSeek-R1 − ChatGPT-4o; n=81; of the original 82 pairs, 1 pair with identical values [tie] was excluded automatically during the Wilcoxon test, resulting in an effective sample size of 81): mean difference=−0.0030; 95% bias-corrected and accelerated CI −0.0110 to 0.005; Wilcoxon W=1440.5000; *z*=−1.0360; *P*=.30; *r*=−0.1150.

### Classification Performance Evaluation

**Figure 2.**
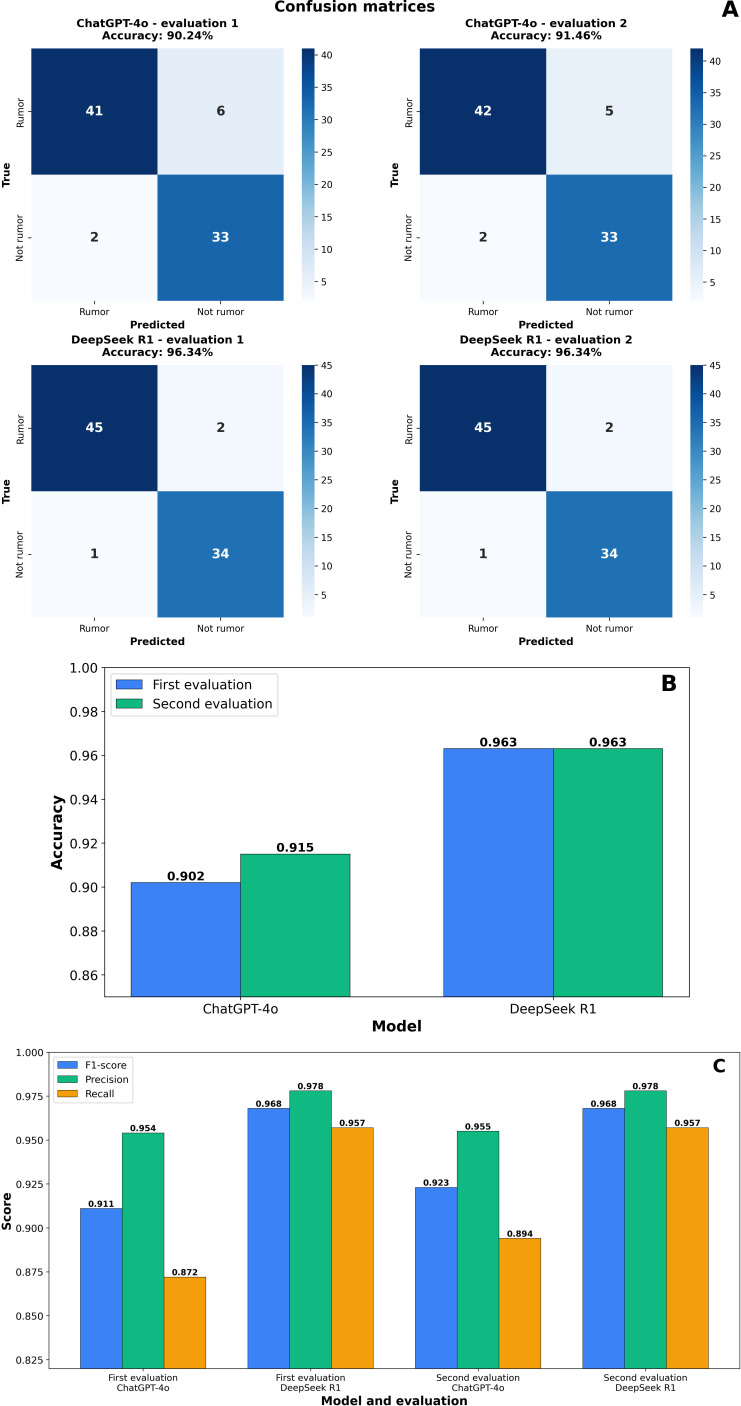
(A) Confusion matrices for vascular lesion classification by ChatGPT-4o and DeepSeek-R1; (B) overall classification accuracy of ChatGPT-4o and DeepSeek-R1; (C) precision, recall, and *F*_1_-scores of ChatGPT-4o and DeepSeek-R1.

Classification performance for hemangioma and vascular malformation statements was evaluated by examining confusion matrices (Figure 2A) and key performance metrics. Confusion matrix analyses indicated no substantial differences in misclassification distribution between the 2 models, with overall good stability. In terms of the overall classification accuracy (Figure 2B), DeepSeek-R1 achieved 0.963, which was notably higher than ChatGPT-4o, which reached approximately 0.910. Additionally, DeepSeek-R1 surpassed ChatGPT-4o in terms of other metrics, including precision, recall, and *F*_1_-score. Specifically, DeepSeek-R1 demonstrated a precision of approximately 0.978, recall of 0.957, and an *F*_1_-score of 0.967, each marginally higher than the corresponding values for ChatGPT-4o (Figure 2C). These results highlight the superior classification accuracy of DeepSeek-R1.

### Expert Rating Analysis

In qualitative assessments, both models demonstrated strong performance regarding the clinical appropriateness of their outputs, with subtle yet meaningful differences observed. Expert ratings (MultimediaAppendices 7  8) indicated that for statements classified as “rumors” (47/82, 57%), DeepSeek-R1 scored significantly higher with a mean (SD) of 4.39 (0.59) and 95% CI 4.21‐4.56 compared to ChatGPT-4o with a mean of 3.96 (SD 0.81) and 95% CI of 3.72‐4.20; the mean difference was 0.431 (95% CI 0.218‐0.644); paired *t*_46_=4.071; *P*<.001; Wilcoxon *P*<.001; and effect size Cohen *d_z_*=0.594.

For statements labeled as “accurate information” (35/82, 43%), DeepSeek-R1 with a mean of 4.44 (SD 0.37) and 95% CI of 4.32‐4.57 also significantly outperformed ChatGPT-4o with a mean of 4.18 (SD 0.69) and 95% CI of 3.94‐4.41; the mean difference was 0.264 (95% CI 0.007‐0.522); paired t_34_=2.085; *P*=.045; Wilcoxon *P*=.046; and Cohen *d_z_*=0.352.

These findings demonstrate significant superiority of DeepSeek-R1 over ChatGPT-4o in evaluating both “rumors” and “accurate information,” with a particularly pronounced advantage in detecting “rumors.”

DeepSeek-R1 performed slightly better than ChatGPT-4o across multiple evaluation dimensions, exhibiting higher output stability and classification accuracy. This finding suggests that DeepSeek-R1 holds greater potential for medical information classification tasks.

## Discussion

This study compared ChatGPT-4o and DeepSeek-R1 in the task of identifying medical rumors, with hemangioma-related misinformation serving as the focal point [17,18]. Overall, both models demonstrated robust language comprehension capabilities but differed markedly in their approaches to recognizing inaccurate statements about hemangiomas. DeepSeek-R1 excelled at pinpointing erroneous claims and clearly categorizing them as rumors, showing its strength in explicit rumor detection and confident classification. In contrast, ChatGPT-4o demonstrated superior semantic similarity and exhibited more consistent stability in understanding nuanced languages, yet tended to approach rumor identification cautiously, often resorting to ambiguous wording rather than decisively refuting false information. Although these observed differences may stem from variations in training data, model architecture, and fine-tuning strategies, existing evidence from other studies suggests that specialized fine-tuning with medical information could further enhance the capability of LLMs in accurately and effectively detecting medical misinformation [19].

In our task, overly cautious responses—specifically, the failure to decisively refute rumors (false negatives)—may perpetuate harmful misconceptions, causing caregivers to delay specialist referrals or discontinue evidence-based treatments in favor of unproven remedies. Conversely, overconfidence—erroneously labeling accurate guidance as rumors (false positives)—may lead to unnecessary anxiety, undermine trust in clinicians, or impede appropriate interventions. In hemangioma treatment, such misclassification could negatively impact decisions regarding timely assessment (eg, ulceration and airway involvement), follow-up intervals, or continuation of guideline-adherent therapies. These risks support the use of conservative safety thresholds, verifiable citations, and escalation of human oversight when model confidence is low. One illustrative example is the claim that “sun exposure exacerbates hemangiomas,” which lacks scientific support [20]. Authoritative sources indicate that sun exposure does not directly enlarge or worsen hemangiomas. While moderate sun protection can help safeguard the skin, it does not specifically address pathological changes in hemangiomas [21,22]. In this study, DeepSeek-R1 correctly identified this assertion as a rumor and provided a concise explanation consistent with medical consensus. ChatGPT-4o, in contrast, did not unequivocally refute the claim, instead offering a somewhat reserved answer that did not effectively dispel the misconception. Although both models possess extensive medical knowledge, DeepSeek-R1 displayed a stronger rumor-debunking ability when confronted with evidently incorrect statements, whereas the cautious approach of ChatGPT-40 diluted its capacity to correct misinformation.

As more users turn to AI assistants for medical information, traditional search engines are gradually being supplemented or even replaced by these systems [23,24]. Unlike search engines that merely provide links, AI chatbots often deliver comprehensive, single-point answers whose perceived authority may lead users to over-rely on them instead of consulting additional information sources [25,26]. Consequently, the adverse impact of inaccurate or ambiguous medical information disseminated by AI could be amplified, posing a considerable risk of misleading patients in their health care decisions. Therefore, ensuring higher accuracy in identifying medical rumors is both urgent and critical [27].

Recent research has proposed various methods for leveraging AI to detect medical rumors. For instance, studies comparing GPT-4 with other models trained specifically on health information have shown that specialized models tend to be more accurate in identifying and correcting misinformation [28,29]. These findings underscore that although LLMs have tremendous potential for conveying medical knowledge, they still exhibit shortcomings in fact-checking and real-time verification [30]. Incorporating real-time retrieval mechanisms and referencing authoritative data in responses represents a key direction for improving the accuracy of AI-generated medical information [28]. Notably, conclusions regarding model superiority depend heavily on the task design, dataset scope, and evaluation criteria. These factors help explain the inconsistencies observed in the existing literature and highlight the novelty of our research, which specifically addresses misinformation related to hemangiomas. The methodological workflow applied in this study—consisting of data annotation, multiround generation, BERT similarity assessment, and expert evaluation—not only validates the relative advantages of DeepSeekR1 in our task but also underscores the insufficiency of any single metric for comprehensively assessing model performance. Multidimensional evaluations more effectively reveal nuanced differences between models in stability, accuracy, and clinical appropriateness, thereby offering valuable lessons and standardized protocols for the deployment and further study of large medical language models.

This study has several limitations. First, our data primarily address hemangiomas and vascular malformations, and the limited number and types of examples may not comprehensively encompass all medical rumors. Second, the labeling of rumors relies on expert judgment, introducing an element of subjectivity, and disagreements may arise when experts evaluate borderline cases. Additionally, discrepancies in the 2 AI models’ training data and knowledge cutoff dates could affect their ability to capture the latest medical information. Finally, we did not evaluate aspects such as explanatory depth, response speed, and user-friendliness. For instance, we did not conduct a formal qualitative or user-centered analysis of explanation quality, which remains an important area for future investigation. For clinical decision support, patient-oriented education, or public health surveillance, LLM-generated outputs should be embedded within regulated workflows that include (1) retrieval-augmented validation from curated vascular anomaly sources, (2) human-in-the-loop review of high-risk recommendations, (3) audit trails and disclaimers clearly delineating accountability, (4) transparent rationales with explicit references to guidelines and clearly marked uncertainties, and (5) postdeployment monitoring for data drift and fairness. These safeguards are prerequisites for mitigating liabilities and improving interpretability and usability in practical applications.

In conclusion, this research highlights the performance differences between the 2 LLMs in detecting hemangioma-related medical rumors, stressing the urgency of maintaining accurate medical information as AI gradually supplants traditional search engines. DeepSeek-R1 showed higher accuracy and a more decisive approach to rumor detection, whereas the guarded stance of ChatGPT-4o sometimes led to less definitive answers. Future studies should optimize AI models’ fact-checking capabilities, for example, by integrating real-time access to authoritative databases, enhancing domain-specific fine-tuning, and building human-machine collaborative monitoring systems. Continuous improvements in the accuracy and transparency of AI-driven medical communications will better protect patient health and reinforce public trust in evidence-based health care.

## Supplementary material

10.2196/76372 Multimedia Appendix 1 Vascular anomaly information sources.

10.2196/76372 Multimedia Appendix 2 Large language model (LLM) text classification prompts.

10.2196/76372 Multimedia Appendix 3 Likert scale for model output assessment.

10.2196/76372 Multimedia Appendix 4 Bidirectional encoder representations from transformers (BERT) semantic similarity code.

10.2196/76372 Multimedia Appendix 5 Bidirectional encoder representations from transformers (BERT) similarity scores model comparison.

10.2196/76372 Multimedia Appendix 6 Bidirectional encoder representations from transformers (BERT) similarity ChatGPT-4o versus DeepSeek-R1.

10.2196/76372 Multimedia Appendix 7 YZ ratings for model reasonableness.

10.2196/76372 Multimedia Appendix 8 WW ratings for model reasonableness.

## Abbreviations

AI: artificial intelligence
BERT: bidirectional encoder representations from transformers
ICC: interclass correlation coefficient
LLM: large language model
