# Supplementary material for: Detection of Medical Misinformation in Hemangioma Patient Education: Comparative Study of ChatGPT-4o and DeepSeek-R1 Large Language Models
Source: JMIR AI. 2025 Nov 18;4:e76372. doi: 10.2196/76372 (PMC12627899; doi:10.2196/76372)
Supplement: Multimedia Appendix 1 [file ai-v4-e76372-s001.docx]

URLs for the information sources and Misconceptions vs. Facts about Hemangiomas and Vascular Anomalies

Social Media Platforms:

- Reddit: <https://www.reddit.com>
- Zhihu: <https://www.zhihu.com>
- Weibo: <https://www.weibo.com>

Medical Education Websites:

- WebMD: <https://www.webmd.com>
- Mayo Clinic: <https://www.mayoclinic.org>
- HaoDaifu: <https://www.haodf.com>

International Medical Guidelines:

- ISSVA (International Society for the Study of Vascular Anomalies): <https://www.issva.org>

**Misconceptions:**

1. "Any red spot or lump on the skin is a hemangioma."
2. "The 'red birthmarks' on newborns' skin are hemangiomas."
3. "Mixed hemangioma is a combination of infantile hemangioma and venous malformation."
4. "Infantile hemangiomas are tumors primarily composed of abnormal capillaries."
5. "Intracranial cavernous hemangiomas are brain tumors that show up on angiography."
6. "Arteriovenous malformations are racemose hemangiomas, which are also a type of hemangioma."
7. "Lymphangiomas are a type of hemangioma and can be treated as hemangiomas."
8. "Hemangiomas are dangerous and could be cancerous."
9. "Untreated hemangiomas will transform into malignant tumors."
10. "If a baby's hemangioma/red spot wasn't present at birth, then it's not a hemangioma."
11. "Hemangiomas only grow on infants; new hemangiomas cannot appear at an older age."
12. "Proper prenatal care or postnatal care can prevent babies from developing hemangiomas."
13. "Children develop hemangiomas only if the pregnant mother was under stress or experienced trauma."
14. "Babies develop birthmarks/hemangiomas in the shape of foods their mothers craved but didn't eat during pregnancy."
15. "Hemangiomas are rare; most red spots on babies' skin are just birthmarks or eczema, not hemangiomas."
16. "Vascular malformations are all congenital; new vascular malformations cannot suddenly occur later in life."
17. "Hemangiomas in children are hereditary; parents with birthmarks or hemangiomas will pass them to their children."
18. "Sun exposure stimulates hemangiomas to grow larger or worsen."
19. "Hemangiomas will disappear on their own by age 1 and won't grow after that."
20. "After a hemangioma recedes on its own, the skin will be completely normal with no trace left."
21. "There is only one type of hemangioma in infants, with no other classifications."
22. "All infantile hemangiomas appear red on the surface and protrude like strawberries."
23. "All hemangiomas will recede on their own; treatment is unnecessary."
24. "Hemangiomas are present from birth and shouldn't be treated; treatment might be harmful."
25. "Infants are too young for treatment; hemangiomas will naturally improve as they grow, so treatment can wait."
26. "Hemangiomas should be surgically removed as soon as discovered; delays can cause major problems."
27. "Surgery is the only effective treatment for hemangiomas."
28. "Hemangioma surgery is a minor procedure with minimal risk."
29. "Never operate on a baby's hemangioma; infants can't tolerate surgery, so use alternative methods instead."
30. "Laser therapy is the first choice for treating hemangiomas; they disappear after one treatment."
31. "Cryotherapy is commonly used to treat hemangiomas; one freezing removes the tumor."
32. "Hormones have such significant side effects that babies with hemangiomas should never be given hormone medications."
33. "I heard eye drops (timolol) can cure a baby's hemangioma."
34. "Is there a therapy that can completely cure hemangiomas in one treatment? There must be an optimal therapy."
35. "All vascular malformations are dangerous and require immediate treatment when discovered."
36. "Vascular malformations only look unappealing but don't affect health."
37. "All types of vascular malformations can be completely cured."
38. "Vascular malformations can only be removed by surgery; other methods are ineffective."
39. "Venous malformations can be cured with traditional Chinese medicine, which is safer than Western medicine."
40. "Once a hemangioma recedes on its own, it's completely cured with no sequelae or need for further treatment."
41. "A certain traditional Chinese doctor/folk remedy can completely cure hemangiomas without recurrence."
42. "Using the newest laser, hemangiomas/port-wine stains can be eliminated in a single treatment."
43. "Certain institutions claim their single therapy can cure all types of hemangiomas."
44. "Advertisements claim hemangioma treatments can be performed without leaving any scars."
45. "Port-wine stains (nevus flammeus) will fade and disappear over time like strawberry hemangiomas."
46. "If a baby has multiple hemangiomas, it's just multiple skin issues."
47. "If a female infant has a hemangioma in the breast area, it doesn't matter; there won't be future effects."

**Correct Information:**

1. Most infantile hemangiomas will partially or completely resolve naturally, but some high-risk hemangiomas require prompt treatment.
2. Not all vascular birthmarks are hemangiomas; according to the ISSVA classification, vascular anomalies include hemangiomas (such as infantile hemangiomas) and vascular malformations (such as port-wine stains), which are fundamentally different in nature and behavior.
3. Infantile hemangiomas typically appear and grow gradually within weeks after birth; red patches present at birth are more likely to be port-wine stains (capillary malformations) or congenital hemangiomas.
4. Port-wine stains are capillary malformations that do not regress spontaneously. They are permanent and may become darker and thicker with age, only lightening with treatments such as laser therapy.
5. What was previously called "lymphangioma" is actually a lymphatic malformation, a benign lesion caused by developmental abnormalities rather than true tumor proliferation, and does not proliferate rapidly like hemangiomas.
6. So-called "cavernous hemangiomas" (such as those in the liver or brain) are actually venous malformations that exist from birth and are not true proliferative hemangiomas.
7. Congenital hemangiomas are fully formed at birth and can be classified as rapidly involuting or non-involuting types; infantile hemangiomas typically appear after birth and grow rapidly during infancy, with a different natural course than congenital hemangiomas.
8. "Cherry hemangiomas" (senile hemangiomas) common in adults are small red spots that appear in adulthood and differ in nature from infantile hemangiomas; infantile hemangiomas primarily occur during infancy.
9. Verrucous hemangioma is a rare congenital capillary/venous malformation present at birth that thickens over time and bleeds easily; angiokeratoma is a more superficial, acquired lesion. They have different clinical presentations and depths and should not be confused.
10. Most infantile hemangiomas can be diagnosed through clinical examination without special imaging; imaging studies like ultrasound or MRI are only considered when the lesion is atypical or suspected to involve deep structures.
11. When an infant has 5 or more cutaneous hemangiomas, internal organs (especially the liver) may also have hemangiomas. Multiple cutaneous hemangiomas (≥5) are an indication for screening infants for hepatic hemangiomas to prevent complications such as cardiac burden from massive liver hemangiomas.
12. Large segmental facial hemangiomas may be part of PHACE syndrome (associated with posterior fossa brain abnormalities, cardiovascular anomalies, eye abnormalities, etc.), requiring comprehensive examinations to rule out these complications.
13. Extensive hemangiomas affecting the lumbosacral or perineal areas may be associated with developmental abnormalities of the vertebrae, spinal cord, or genitourinary system (such as LUMBAR or PELVIS syndrome). Therefore, these lesions require spinal and abdominal ultrasound examinations to screen for potential malformations.
14. Periocular hemangiomas may obstruct or compress the eyeball, leading to vision development problems (such as ptosis causing amblyopia, corneal astigmatism, etc.). These are high-risk lesions requiring early intervention to protect vision.
15. Infantile hemangiomas follow a natural growth pattern: most begin to appear 1-2 weeks after birth, proliferate rapidly at 1-5 months, reaching about 80% of their maximum volume around 5 months; growth then tends to stop, followed by a slow regression phase, generally beginning to shrink and fade in late infancy.
16. Whether a hemangioma causes complications depends not only on size but also on location and type. Some small hemangiomas in critical locations (such as eyelids, lips) can cause serious problems, while small hemangiomas on the trunk usually pose lower risks.
17. Some hemangiomas are located deep in the skin (called deep hemangiomas) and appear not as bright red patches but as skin-colored or bluish-purple subcutaneous protrusions; there are also mixed-type hemangiomas with both superficial and deep components.
18. Whether skin changes remain after hemangioma regression depends on its characteristics. Larger diameter, significantly raised (>2 mm above skin level), or steep-bordered hemangiomas are more likely to leave persistent skin changes (such as scarring, skin atrophy, or telangiectasia).
19. According to the latest guidelines, the first-line treatment for infantile hemangiomas is oral propranolol (a β-blocker), not corticosteroids. Systemic steroids are only considered as second-line treatment when propranolol is contraindicated or ineffective.
20. Under close medical supervision, propranolol treatment for infantile hemangiomas is safe and effective. This therapy has been extensively studied and approved by regulatory authorities, and is considered the standard treatment for complex hemangiomas.
21. If an infantile hemangioma does not respond to propranolol or propranolol cannot be used, other therapies may be considered. For example, oral or local injection of corticosteroids can be used to reduce the lesion, and for special severe cases, newer medications (such as sirolimus) are also available.
22. For small superficial hemangiomas, topical β-blockers (such as timolol gel) are one of the effective treatments. Guidelines recommend timolol for superficial or thin hemangiomas to reduce their growth and redness.
23. Laser therapy (such as pulsed dye laser) is mainly used to accelerate healing of ulcerated hemangiomas or to remove residual red marks after complete regression. For proliferating hemangiomas, laser has limited effect on reducing tumor mass and is usually not used as a first-line treatment for tumor reduction.
24. For non-emergency hemangiomas, it is usually recommended to wait for natural regression during the infantile period before electively removing residual lesions surgically, which can reduce the scope of surgery and achieve better cosmetic results. Early surgical intervention before hemangioma regression is only considered when life-threatening or severely affecting function.
25. After discontinuing treatments such as propranolol, some infantile hemangiomas may experience a 'rebound' regrowth. According to reports, approximately 6%-25% of cases show partial regrowth within months after stopping medication, requiring re-intervention or continued follow-up observation based on the situation.
26. If significant skin problems remain after hemangioma regression (such as dilated superficial vascular networks, loose excess skin, or scar tissue), they can be improved through laser treatment or surgical reconstruction when the child is older to restore a more normal appearance.
27. Infantile hemangiomas are benign proliferations of vascular tissue and do not transform into malignant tumors. Their risks mainly lie in local growth, compression, ulceration, and other complications, not 'cancerous transformation'.
28. Many infantile hemangiomas tend to complete regression before school age (about 4-5 years), but nearly half of the children may retain some skin changes or tissue residuals. Some hemangiomas have not completely disappeared by school age and require treatment decisions based on the residual condition.
29. The exact cause of infantile hemangiomas remains unclear. Research has not found clear associations between maternal diet, environment, or behavior and the occurrence of hemangiomas, nor has a single genetic mutation been identified that directly causes hemangiomas.
30. Certain infant populations have a higher risk of developing infantile hemangiomas. For instance, female infants are more likely to have hemangiomas than males, and premature or low birth weight infants, twins or multiple births, and Caucasian infants also have higher incidence rates.
31. Even smaller or superficial hemangiomas, if located in areas prone to friction or moisture (such as lips, neck skin folds, or diaper areas), may ulcerate and bleed. Close observation and enhanced skin care should be maintained in these high-risk areas.
32. Research shows that propranolol, as a first-line medication for treating infantile hemangiomas, has good safety. Most infants experience only minor adverse reactions, severe side effects are very rare, and even when they occur, they can usually be reversed through measures such as discontinuing the medication.
33. Not all hemangiomas require treatment. Approximately 80%-90% of infantile hemangiomas are small in volume, not in sensitive locations, and will not cause problems; they can regress on their own under observation without intervention. Only those hemangiomas at risk of complications or likely to leave significant deformities need treatment.
34. The most common sites for infantile hemangiomas are the head and face, followed by the trunk and limbs. About 60% of hemangiomas occur in the head and neck region, possibly related to the abundant neural crest-derived vascular progenitor cells in these areas. Relatively speaking, the probability of hemangiomas appearing in other parts of the body is slightly lower.
35. Hemangiomas occurring in the breast area of female infants are considered high-risk locations because they may affect the normal development of breast tissue and nipples. For hemangiomas in this area, doctors typically follow up more aggressively or even treat them to avoid future breast development deformities.
