# Supplementary material for: Detection of Medical Misinformation in Hemangioma Patient Education: Comparative Study of ChatGPT-4o and DeepSeek-R1 Large Language Models
Source: JMIR AI. 2025 Nov 18;4:e76372. doi: 10.2196/76372 (PMC12627899; doi:10.2196/76372)
Supplement: Multimedia Appendix 2 [file ai-v4-e76372-s002.docx]

Multimedia Appendix 2

Prompts for Text Classification by Large Language Models

Evaluate the following statement for accuracy and reliability in the context of hemangioma and vascular malformation treatment:
