# Supplementary material for: Detection of Medical Misinformation in Hemangioma Patient Education: Comparative Study of ChatGPT-4o and DeepSeek-R1 Large Language Models
Source: JMIR AI. 2025 Nov 18;4:e76372. doi: 10.2196/76372 (PMC12627899; doi:10.2196/76372)
Supplement: Multimedia Appendix 3 [file ai-v4-e76372-s003.docx]

**Multimedia Appendix 3: Expert Evaluation Likert Scale for Model Output Assessment**

| **Score** | **Label** | **Description** |
| --- | --- | --- |
| 1 | Highly Inconsistent | The output is extremely illogical, contradictory, or irrelevant. It does not address the prompt adequately and exhibits multiple severe flaws. |
| 2 | Some Inconsistencies | The output contains partial or superficial correctness but has notable flaws and inconsistencies. It may miss important points or introduce errors. |
| 3 | Moderately Consistent | The output is mostly reasonable and addresses the prompt, though some inaccuracies or gaps are present. Further refinement would be needed. |
| 4 | Mostly Consistent | The output is generally accurate, coherent, and relevant. Minor errors or omissions may exist, but overall, it aligns well with the prompt. |
| 5 | Very Reasonable | The output is fully coherent, accurate, and addresses all critical aspects of the prompt with no major errors. It demonstrates strong clarity overall. |

*Description:* This table details the 5-point Likert scale rubric (from 1 = "highly inconsistent" to 5 = "very reasonable") used by experts to score model outputs.
