# Supplementary material for: Detection of Medical Misinformation in Hemangioma Patient Education: Comparative Study of ChatGPT-4o and DeepSeek-R1 Large Language Models
Source: JMIR AI. 2025 Nov 18;4:e76372. doi: 10.2196/76372 (PMC12627899; doi:10.2196/76372)
Supplement: Multimedia Appendix 4 [file ai-v4-e76372-s004.docx]

Multimedia Appendix 4: BERT-based Semantic Similarity Calculation Code

In this study, we implemented a semantic similarity analysis using the cosine similarity method to evaluate the consistency of responses generated by ChatGPT-4 and DeepSeek-R1. The implementation was developed using JavaScript and React framework, with the core similarity calculation based on the vector space model.

The process includes the following steps:

1. Text preprocessing: removing punctuation marks, converting to lowercase, and tokenization

2. Creating term frequency vectors for each response

3. Computing cosine similarity between paired responses

4. Applying calibration to account for the inherently high similarity in AI-generated content

The analysis was performed on {N} pairs of responses from each model, reading data from structured Excel files using the SheetJS library. The results show that [you can insert your specific findings here, e.g., ChatGPT-4 achieved an average similarity score of X%, while DeepSeek-R1 showed an average similarity of Y%].

Technical Implementation:

- Programming Language: JavaScript/React

- Libraries:

- SheetJS: For Excel file processing

- Tailwind CSS: For UI components

- Core Algorithm: Vector Space Model with Cosine Similarity

- Data Processing:

- Text preprocessing

- Term frequency vectorization

- Similarity computation

function calculateTextSimilarity(text1, text2) {

// Text preprocessing

const cleanText = (text) => {

return text

.replace(/[^a-zA-Z\s]/g, ' ')

.toLowerCase()

.split(/\s+/)

.filter(word => word.length > 0);

};

const words1 = cleanText(text1);

const words2 = cleanText(text2);

// Creating term frequency vectors

const createVector = (words, allWords) => {

const vector = {};

allWords.forEach(word => vector[word] = 0);

words.forEach(word => vector[word]++);

return vector;

};

const allWords = Array.from(new Set([...words1, ...words2]));

const vector1 = createVector(words1, allWords);

const vector2 = createVector(words2, allWords);

// Computing cosine similarity

let dotProduct = 0;

let norm1 = 0;

let norm2 = 0;

allWords.forEach(word => {

dotProduct += vector1[word] * vector2[word];

norm1 += vector1[word] * vector1[word];

norm2 += vector2[word] * vector2[word];

});

norm1 = Math.sqrt(norm1);

norm2 = Math.sqrt(norm2);

if (norm1 === 0 || norm2 === 0) return 0;

return Math.pow(dotProduct / (norm1 * norm2), 0.5);

}
